# In This Issue

**Published:** 1999

## ALCOHOLISM TREATMENT IN THE UNITED STATES: AN OVERVIEW

Each day, more than 700,000 people in the United States receive treatment for alcoholism. Numerous inpatient and outpatient treatment options are available to those patients. Commonly used treatment components include detoxification; cognitive-behavioral therapy; motivational enhancement therapy; 12-step programs, such as Alcoholics Anonymous; and pharmacotherapy. These components can be used as stand-alone treatments or in combination. Drs. Richard K. Fuller and Susanne Hiller-Sturmhöfel review these treatment approaches as well as studies comparing their effectiveness. For example, research shows that certain approaches (e.g., 12-step facilitation therapy) may be best suited for patients with specific characteristics (e.g., more severe alcohol dependence).

## COGNITIVE-BEHAVIORAL COPING-SKILLS THERAPY FOR ALCOHOL DEPENDENCE

To improve a patient’s skills for changing problem behaviors (e.g., excessive alcohol consumption), therapists frequently use cognitive-behavioral coping-skills therapy (CBST), particularly in academic and Veterans Affairs hospitals. Though widely used, CBST is not well defined. In this article, Drs. Richard Longabaugh and Jon Morgenstern summarize results from studies attempting to identify the active components of CBST. In reviewing studies on the effectiveness of CBST, the authors were unable to find specific CBST characteristics that could account for the treatment’s overall effectiveness. Furthermore, CBST appears to be more effective than other treatments, but only when added to ongoing treatment and not when delivered as stand-alone therapy.

## MOTIVATION FOR CHANGE AND ALCOHOLISM TREATMENT

Motivation plays an important role in alcoholism treatment, enabling patients to initially seek treatment, comply with and complete a treatment regimen, and maintain successful long-term changes in their drinking patterns. Researchers have outlined a series of “stages of change” to describe the motivational steps that a person must take to change his or her behavior. Such steps enable clinicians and researchers to design more effective treatment programs that are geared to help motivate unmotivated patients. Dr. Carlo C. DiClemente, Ms. Lori E. Bellino, and Ms. Tara M. Neavins explore the role of motivation in treatment and describe the motivational treatment approaches known as brief intervention, motivational interviewing, and motivational enhancement therapy.

## FACILITATING 12-STEP SELF-HELP GROUP INVOLVEMENT

Facilitating patients’ involvement with 12-step self-help organizations, such as Alcoholics Anonymous (AA) and Narcotics Anonymous (NA), often is the chief goal of substance abuse treatment. According to Dr. Keith Humphreys, 12-step-facilitation (TSF) interventions are consistent with AA’s principles and practices and are designed to increase patients’ 12-step group involvement and promote abstinence. Dr. Humphreys presents the rationale for using TSF interventions and reviews research comparing TSF interventions with other treatment methods.

## MEDICATIONS TO TREAT ALCOHOLISM

Advances in neuroscience have helped identify many of the mechanisms underlying addiction, paving the way for the development of medications that can assist in alcoholism therapy. Drs. Bankole A. Johnson and Nassima Ait-Daoud explore the use of recently introduced and experimental medications that act within the brain to reduce the desire to drink and to promote abstinence. Researchers currently are evaluating the efficacy of naltrexone (i.e., ReVia™), a medication recently approved for alcoholism treatment, and acamprosate. Another medication, ondansetron, shows promise in treating the early onset of alcoholism, a period during which people tend to respond poorly to conventional psychosocial treatment. The authors explain the need for future research on the effects of individual medications on different subtypes of alcoholism and the interactions between pharmacological and psychosocial treatment approaches.

## COPING-SKILLS TRAINING AND CUE-EXPOSURE THERAPY IN ALCOHOLISM TREATMENT

Coping-skills training (CST) aims to build and enhance an alcoholic patient’s skills so that he or she can resist the urge to drink. An extension of CST, cue-exposure therapy (CET) exposes the patient to alcohol-related cues (e.g., the smell of alcohol) in a treatment setting, thereby allowing the patient to practice his or her coping skills. Both of these treatment approaches are relatively new additions to the armamentarium of alcoholism treatment providers. Drs. Peter M. Monti and Damaris J. Rohsenow explore the conceptual bases, treatment methods, and effectiveness of both CST and CET. Various studies have indicated that CST can substantially improve treatment outcome when added to other comprehensive treatment programs. Existing analyses of the CET approach also have shown promising results.

## THE COMMUNITY-REINFORCEMENT APPROACH

The community-reinforcement approach (CRA) to alcoholism treatment is twofold: It provides incentives to the patient to stop him or her from drinking and seeks to eliminate the pleasurable aspects of alcohol consumption. For example, CRA therapists strive to build the patient’s motivation to quit drinking; help him or her initiate abstinence; and increase positive reinforcement by helping the patient develop rewarding social, occupational, and recreational relationships and activities. As Drs. William R. Miller and Robert J. Meyers report, numerous studies have demonstrated that CRA is more effective than other treatment approaches. Furthermore, CRA offers a highly flexible treatment approach that can be adapted to a wide variety of patient populations.

## CONTINGENCY MANAGEMENT

Contingency management (CM) is a strategy used in the treatment of alcohol and other drug (AOD) abuse to encourage abstinence and other treatment-related goals by rewarding good behavior while punishing undesired behavior. Patients who demonstrate abstinence are given positive reinforcers, such as vouchers for retail goods. Such reinforcers are withheld when patients exhibit AOD use. Drs. Stephen T. Higgins and Nancy M. Petry discuss findings from research with animals that provide the conceptual background for CM. The authors also review findings from human studies that demonstrate the effectiveness of CM interventions in reducing AOD use; improving treatment attendance; and reinforcing other treatment goals, such as compliance with a medication regimen or obtaining employment.

## BRIEF INTERVENTION IN PRIMARY CARE SETTINGS

Primary care providers play an important role in identifying and initiating treatment for patients with alcohol problems. One treatment approach that has been shown to be effective in this setting is physician-delivered brief intervention, report Dr. Michael Fleming and Ms. Linda Baier Manwell. Brief intervention generally consists of assessment and feedback, negotiation and goal setting, behavior-modification techniques, bibliotherapy, and followup and reinforcement. Despite evidence supporting the effectiveness of brief intervention, however, primary care providers have not yet widely implemented this approach, at least in part because of lack of adequate training and the complexities of the current health care system.

## TREATING PROBLEM DRINKING

A person may have a problem with alcohol without being an alcoholic. In fact, according to recent data, most people have only mild-to-moderate alcohol problems. Relative to alcoholics, these drinkers have a shorter problem-drinking history, more social and economic stability, and greater personal resources. In this article, Drs. Kimberly S. Walitzer and Gerard J. Connors describe a two-step treatment approach for problem drinkers. The first step is to make the drinker aware of the situations that trigger his or her desire to drink. The second step is to help the drinker change his or her drinking behavior when confronted with those situations. This cognitive-behavioral approach is designed specifically for problem drinkers who want to reduce their drinking and who do not have a strong physical dependence on alcohol. The article includes descriptions of various drinking-reduction techniques and reviews evidence that supports the need for drinking-reduction training.

## TREATING ALCOHOL-DEPENDENT PATIENTS WHO HAVE PSYCHIATRIC DISORDERS

Psychiatric disorders occur more frequently among persons who abuse or who are dependent on alcohol than among people in the general population. Alcoholics with co-occurring psychiatric conditions may be more susceptible to psychosocial problems and less likely to achieve and maintain abstinence than are alcoholics without such disorders. In addition, heavy drinking may produce or worsen symptoms of depression or anxiety. The evaluation of psychiatric symptoms in alcoholic patients is difficult because of the complexity of the relationships among heavy drinking, psychiatric symptoms, and personality factors. Drs. Vania Modesto-Lowe and Henry R. Kranzler describe research on the link between alcohol-related and psychiatric disorders. The authors also suggest general therapeutic considerations as well as treatment strategies targeting specific comorbidities.

## RELAPSE PREVENTION: AN OVERVIEW OF MARLATT’S COGNITIVE-BEHAVIORAL MODEL

Successful alcoholism treatment hinges on preventing a relapse to drinking, which can occur even after prolonged abstinence. Dr. Mary E. Larimer, Ms. Rebekka S. Palmer, and Dr. G. Alan Marlatt review a key relapse prevention (RP) model that has been widely used in alcoholism treatment in recent years. The model posits that several factors may contribute to relapse. Particularly high-risk situations include being anxious or euphoric as well as receiving pressure from peers to take a drink. The RP model reviewed here incorporates a variety of intervention strategies that are designed to target each step in the relapse process. The authors also present the results of studies that examine the effectiveness of the RP approach.

